# Supplementary material for: Biochemical Screening of Phytochemicals and Identification of Scopoletin as a Potential Inhibitor of SARS-CoV-2 Mpro, Revealing Its Biophysical Impact on Structural Stability
Source: Viruses. 2025 Mar 12;17(3):402. doi: 10.3390/v17030402 (PMC11945487; doi:10.3390/v17030402)
Supplement: Supplementary file 1 [file viruses-17-00402-s001.zip › viruses-3448217-supplementary.pdf]

viruses

MDPI

Supplementary material for Research article

Biochemical Screening of Phytochemicals and Identification of Scopoletin as a Potential Inhibitor of SARS-CoV-2 M^{pro}, Revealing Its Biophysical Impact on Structural Stability

Sarika Bano [1,†], Jyotishna Singh [1,†], Zainy Zehra [2], Md Nayab Sulaimani [2], Taj Mohammad [2], Seemasundari Yumlembam [3], Md Imtaiyaz Hassan [2], Asimul Islam [2] and Sanjay Kumar Dey [1,*]

[1] Laboratory for Proteins and Structural Biology, Dr. B.R. Ambedkar Center for Biomedical Research, University of Delhi, Delhi 110007, India; sbano@acbr.du.ac.in (S.B.); jyotishnasingh08@gmail.com (J.S.)
[2] Centre for Interdisciplinary Research in Basic Sciences, Jamia Millia Islamia, Jamia Nagar, New Delhi 110025, India; zainyzehrajmi@gmail.com (Z.Z.); md186547@st.jmi.ac.in (M.N.S.); taj144796@st.jmi.ac.in (T.M.); mihassan@jmi.ac.in (M.I.H.); aislam@jmi.ac.in (A.I.)
[3] Laboratory for Proteins, Dr. B.R. Ambedkar Center for Biomedical Research, University of Delhi, Delhi 110007, India; seemasundari@gmail.com
* Correspondence: sdey@acbr.du.ac.in; Tel.: +919205337595
† These authors contributed equally to this work.

Supplementary material:

1. Characterisation Data of Native M^{pro}

1.1 UV Spectroscopy

UV spectroscopy was performed to calculate the concentration of purified protein. It was also used to assess the quality and folding of protein. In the graph plotted against wavelength vs absorbance, a peak was observed at 280 nm with an absorbance of 0.6 **(Figure S1)**. Using this absorbance value the concentration of purified M^{pro} protein was calculated as per the Beer Lambert Law. The calculated concentration of protein was 0.6 mg/mL (18 μM).

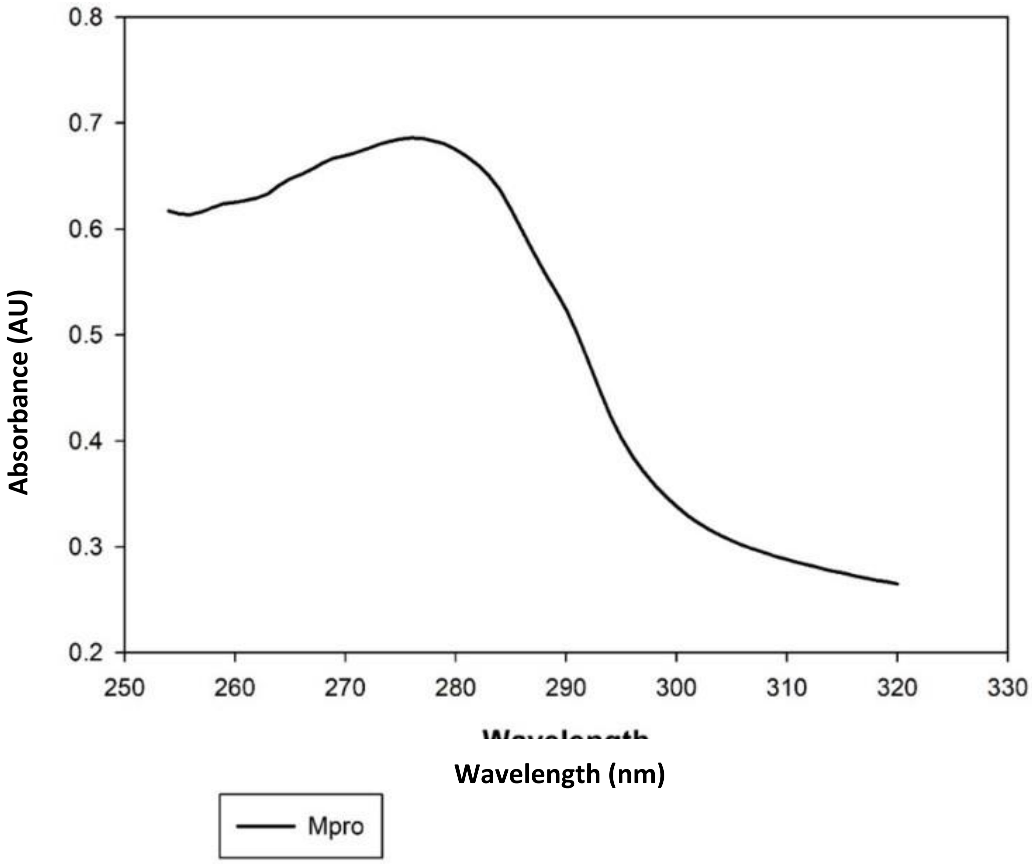

Figure S1. UV-vis absorption spectrum of SARS-CoV-2 M^{pro}. The spectrum was recorded in the range of 250-330 nm, showing a characteristic peak around 280 nm corresponding to aromatic amino acids present in the protein with an absorbance of 0.6.

1.2 Florescence Spectroscopy

Fluorescence of the native M^{pro} protein was measured to gain an insight on its tertiary structure and ensure that it has folded correctly after purification. A correctly folded protein typically shows a peak around 333 nm due to tryptophan residues. A peak was observed at 336 nm on the graph plotted between wavelength and intensity indicating that the protein is correctly folded **(Figure S2).**

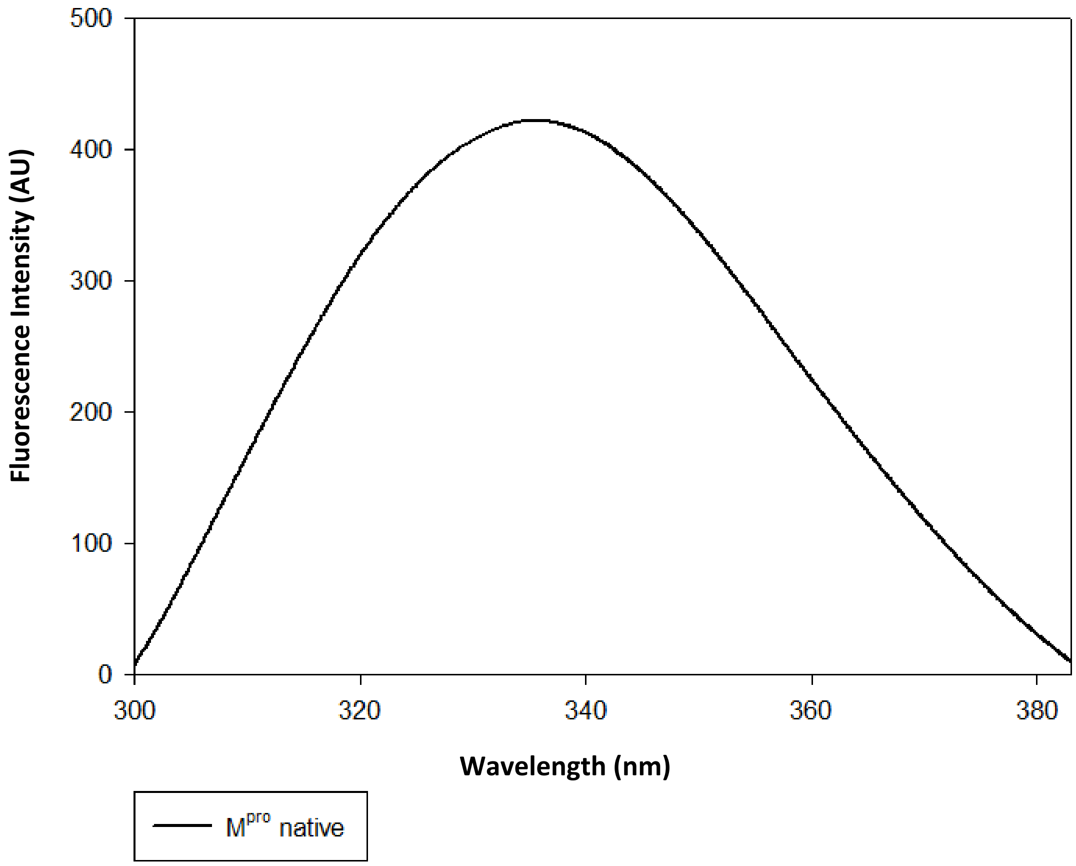

Figure S2. Fluorescence emission spectrum of SARS-CoV-2 M^{pro}, recorded at an excitation of 292 nm. A peak at 336 nm corresponds to the intrinsic fluorescence of tryptophan residues in the native state of the protein.

1.3 CD Spectroscopy

CD Spectroscopy was performed to analyse the secondary structure of SARS-CoV-2 M^{pro}. The readings were taken in Far-UV range (185-250 nm). Two peaks were observed, one around 208 nm and other around 222 nm which indicated that alpha helix is more prominent in the M^{pro} protein structure **(Figure S3).**

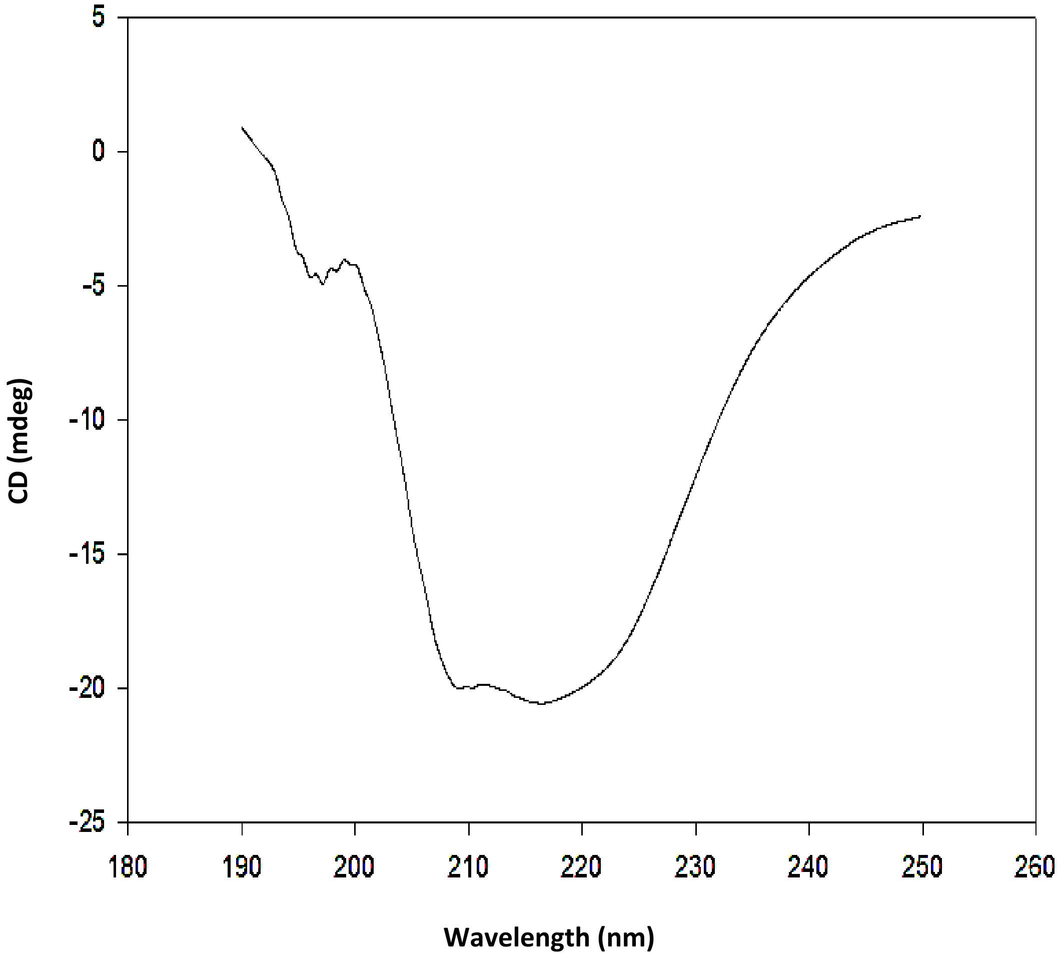

Figure S3. Far-UV CD spectrum of SARS-CoV-2 M^{pro} showing two prominent peaks at 208 nm and 222 nm indicating that alpha-helical structures are prominent in the protein.

1.4 Thermal Denaturation

Circular dichroism (CD) spectra were recorded in a JASCO-CD POLARIMETER at 25 °C. Far-UV spectrum was recorded at wavelengths between 190 and 260 nm in a 0.1 cm path-length cuvette. Near-UV CD spectrum was recorded between 250 and 310 nm in a 1 cm path-length cuvette. Thermal denaturations were monitored by CD, employing a protein concentration of 20 µM and performing thermal denaturation from the temperature 20°C to 80°C **(Figure S4).**

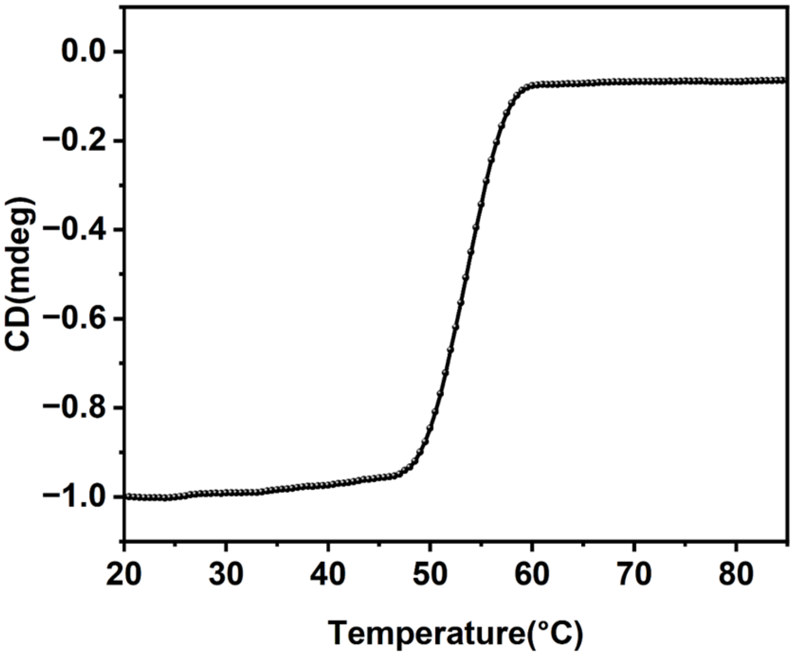

Figure S4: A graph representing thermal melting temperature at which protein denatures. The calculated Tm was 45°C.

2. MTT Assay

The cytotoxic effects of Scopoletin were evaluated using the MTT assay, where Quercetin served as a control. Cell viability percentage was assessed at concentrations ranging from 0 to 100 μM of ligands. The dose-response graph demonstrated that Scopoletin maintained cell viability and did not show any significant cytotoxicity, particularly at lower concentrations (10-50 μM) **(Figure. S6).** The viability did decrease slightly but remained comparable to Quercetin, indicating favourable safety profile for Scopoletin even at elevated doses.

The control, Quercetin, also maintained a consistent and high cell viability throughout all tested concentrations, thus serving as a benchmark for comparison. The overall findings indicated that Scopoletin successfully maintains the acceptable cell viability, comparable to that of the control, Quercetin, and therefore supports its potential as a viable therapeutic candidate for further evaluation. The HEK293 cells pre and post treatment with Scopoletin can be seen in **Figure S5 (a) and S5 (b),** respectively.

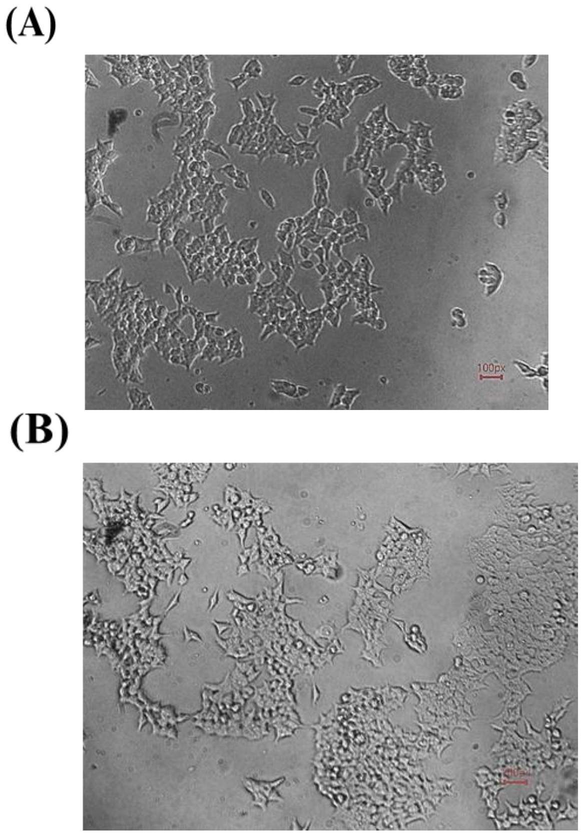

Figure S5. HEK293 Cells before and after treatment of Scopoletin. - **(a)** HEK293 Cells before drug treatment, and **(b)** after 24 h with drug treatment.

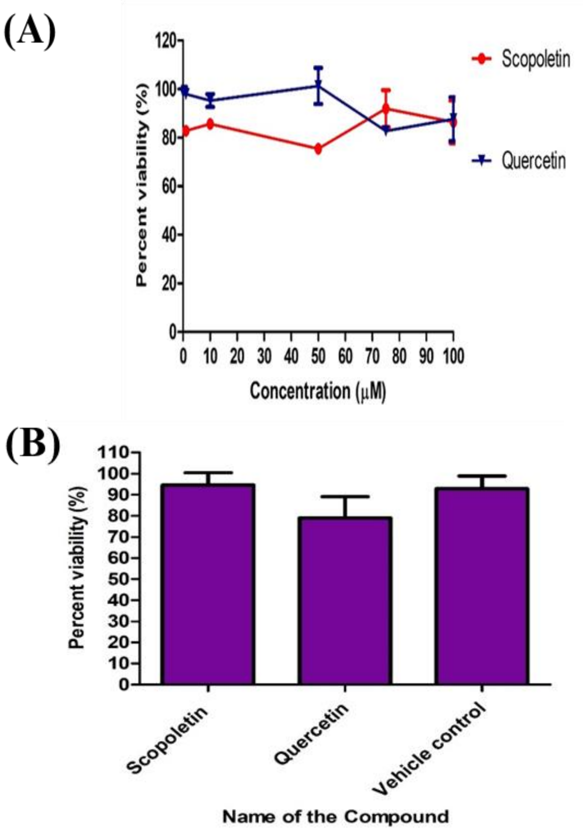

Figure S6. Phytochemical cytotoxicity effect with different concentrations of drug. **(a)** Percent Viability graph versus concentration, **(b)** Percent Viability graph versus name of the compounds.

3. FRET- based enzyme Assay in presence of Quercetin

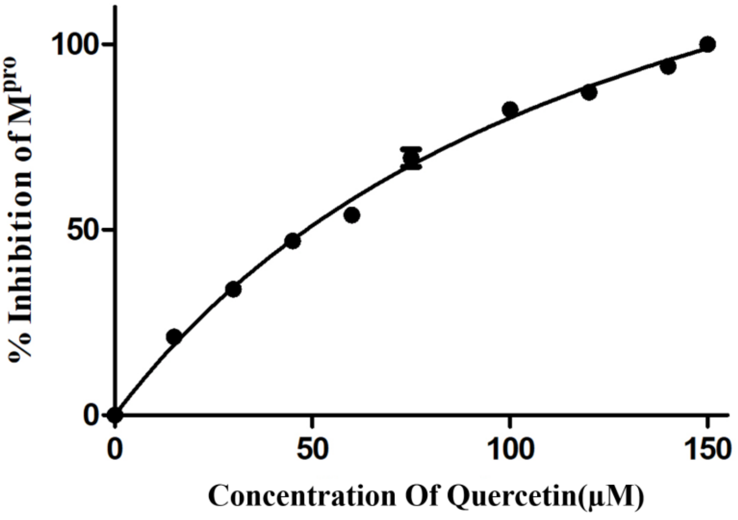

Figure S7. Graph showing dose-dependent inhibition of M^{pro} with % inhibition plotted against quercetin concentration. The IC_{50} of Quercetin for Mpro was calculated as 49.6 μM.

4. Ac-Abu-Tle-Leu-Gln-MCA Substrate details

Peptide Institute, Inc.
7-2-9 Saito-Asagi, Ibaraki-Shi, Osaka 567-0085, Japan

Safety Data Sheet (SDS)

1. PRODUCT AND COMPANY IDENTIFICATION

Code Number: 3250-v
Product Name: Ac-Abu-Tle-Leu-Gln-MCA
Supplier's Name: Peptide Institute, Inc.
Address: 7-2-9 Saito-Asagi, Ibaraki-Shi, Osaka 567-0085, Japan
Phone Number: 81-72-643-4411
Fax Number: 81-72-643-4422
Recommended uses: Reagent
Restrictions on use: Seek expert judgment as necessary.
Creation Date: April 14, 2020
Revised: March 12, 2024 (ver.3)

2. HAZARDS IDENTIFICATION

Classification of the substance or mixture
GHS classification Not a hazardous substance
Other hazards: The chemical, physical and toxicological properties of this product have not been thoroughly investigated. Exercise due care.

3. COMPOSITION/INFORMATION ON INGREDIENTS

Single Substance or Mixture: Single Substance
Common Chemical Name, Common Name or Substance Name:
Acetyl-L-α-aminobutyroyl-L-*tert*-leucyl-L-leucyl-L-glutamine α-(4-methylcoumaryl-7-amide)
Molecular Formula: $C_{33}H_{48}N_6O_8$ (M.W. 656.77)
Product Description: Fluorogenic Substrate for SARS-CoV/SARS-CoV-2 M^{pro} (a.k.a. $3CL^{pro}$)
CAS Registry Number: -
EINECS No.: -
UN No. & Hazard Class: -

Figure S8. Details of substrate used for FRET-based enzymatic assay procured from **Peptide Institute, Inc., Japan.**

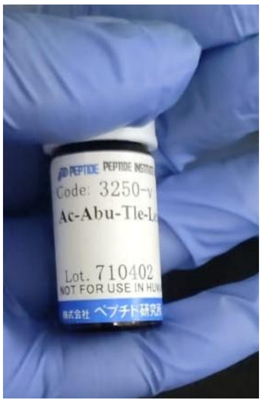

Figure S9. Image of substrate vial used for FRET-based assay with visible Lot no.

5. Docking Results of Scopoletin with Mpro of other Family

6.1. Mpro SARS-CoV-1- PBD ID: 7ZQW

Name of the ligand	Binding Free Energy (kcal/mol)
Scopoletin	-5.1

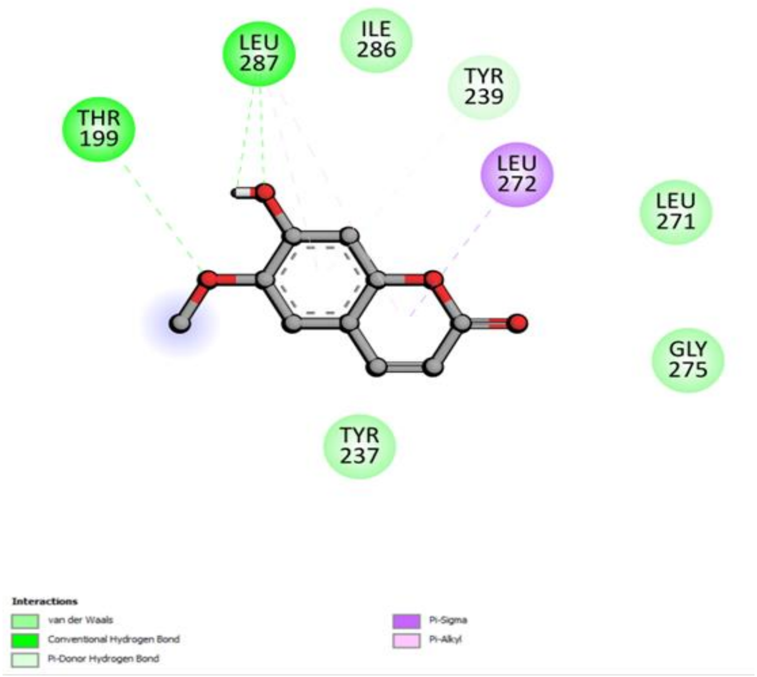

Figure S10. 2D interaction study of SARS-CoV-1 Mpro with the Scopoletin**,** the conventional hydrogen bond are farmed with the Scopoletin at position **THR:199** and **LEU:287**.

6.2.Mpro MERS-CoV PBD ID: 9BOO

Name of the ligand	Binding Free Energy (kcal/mol)
**Scopoletin**	**-6.7**

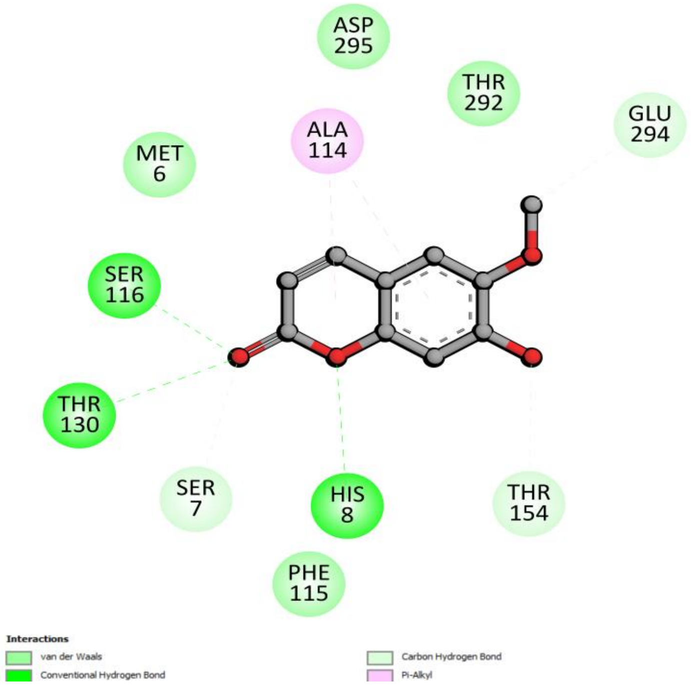

Figure S11. 2D interaction study of MERS-CoV Mpro with the Scopoletin**,** the conventional hydrogen bond are farmed with the Scopoletin at position **SER:116 ,THR:130 and HIS:8.**
